# Can Transthoracic Echocardiography Be Used to Predict Fluid Responsiveness in the Critically Ill Patient? A Systematic Review

**DOI:** 10.1155/2012/513480

**Published:** 2012-02-06

**Authors:** Justin C. Mandeville, Claire L. Colebourn

**Affiliations:** Adult Intensive Care Unit, John Radcliffe Hospital Oxford, Headley Way, Headington, Oxford OX3 9DU, UK

## Abstract

*Introduction*. We systematically evaluated the use of transthoracic echocardiography in the assessment of dynamic markers of preload to predict fluid responsiveness in the critically ill adult patient. *Methods*. Studies in the critically ill using transthoracic echocardiography (TTE) to predict a response in stroke volume or cardiac output to a fluid load were selected. Selection was limited to English language and adult patients. Studies on patients with an open thorax or abdomen were excluded. *Results*. The predictive power of diagnostic accuracy of inferior vena cava diameter and transaortic Doppler signal changes with the respiratory cycle or passive leg raising in mechanically ventilated patients was strong throughout the articles reviewed. Limitations of the technique relate to patient tolerance of the procedure, adequacy of acoustic windows, and operator skill. *Conclusions*. Transthoracic echocardiographic techniques accurately predict fluid responsiveness in critically ill patients. Discriminative power is not affected by the technique selected.

## 1. Introduction

Our primary concern in the management of the critically ill patient is the optimisation of tissue oxygen delivery. Insufficient intravascular loading in the early resuscitation of acute sepsis results in tissue underperfusion, organ dysfunction, and acidosis. Excessive fluid administration has also been shown to be detrimental in the perioperative setting and in acute lung injury, prolonging both time on mechanical ventilation and time in intensive care [1].

It has been reported that as few as 40 percent of critically ill patients thought to be intravascularly deplete gain an improvement in cardiac output after a standard fluid bolus, exposing more than half of patients to the risks of excessive fluid administration [2].

Knowledge of static measures of preload such as central venous pressure, pulmonary artery wedge pressure, end-diastolic volumes, and intrathoracic blood volume has not translated into patient benefit [3–6]. This suggests that measurement of preload does not foretell preload responsiveness.

Contemporary investigation has therefore focussed on the search for clinical markers which predict a useful response to a fluid bolus. These “dynamic” markers make use of provoked cardiac reaction assessed without the need for a fluid bolus, instead utilizing either the consequences of heart-lung interaction during ventilation or the response to postural change to mimic the effect of a fluid bolus on stroke volume.

**Figure 1:**
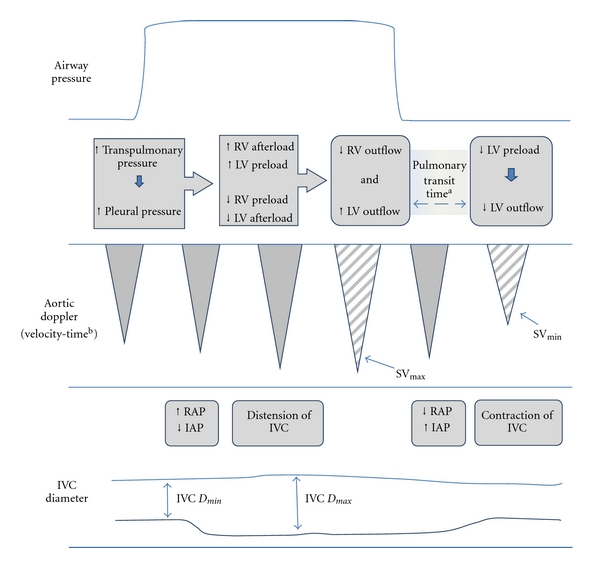
The physiological explanation for the changes in stroke volume and IVC diameter caused by mechanical ventilation. RV right ventricle, LV left ventricle, SV_max⁡_ and SV_min⁡_ maximum and minimum stroke volume, RAP right atrial pressure, IAP intraabdominal pressure, IVC *D*_max⁡_ and IVC_min⁡_ maximum and minimum inferior vena cava diameter during the cycle. ^a^The pulmonary transit time represents the time taken for blood to travel through the pulmonary circulation. ^b^SV is the product of the velocity-time integral (area under the Doppler signal curve) and the diameter of the vessel at the point the reading was taken.

Firstly, in mechanically ventilated patients who have no spontaneous respiratory effort, the change in intrathoracic pressure has a cyclical effect on both the left and right heart (as shown in Figure 1). A rise in intrapleural pressure compresses the pulmonary vasculature and in turn causes compression of the venous inflow vessels and the heart itself. This reduces both right ventricular (RV) preload and left ventricular (LV) afterload whilst conversely both RV afterload and LV preload are increased. These effects are accentuated by hypovolaemia implying variation in stroke volume with cyclical respiratory changes can be used to predict whether stroke volume will alter if preload is increased. This is the basis of the increasingly ubiquitous stroke volume variation monitoring systems but can also be examined using Doppler echocardiography of flow through valves, vessels, or outflow tracts. If the cross-section at the point of measurement can be visualized or accurately estimated, then the product of that area and the integral of the flow-time curve (generated by the Doppler signal) is equal to the stroke volume.

Secondly, in the spontaneously ventilating subject, negative intrapleural pressure during inspiration results in a reduction in the diameter of the abdominal inferior vena cava (IVC). The degree of collapse during tidal volume breaths is known to reflect the right atrial pressure with reasonable accuracy in health [2]. To a degree, the reverse effect is seen in patients who are being ventilated with positive pressure.

Thirdly, raising the legs from the horizontal position to 45 degrees causes the gravitational movement of lower limb venous blood towards the heart. This provides a transient volume load of between 150 and 300 millilitres to the central circulation, lasting for a few minutes [7, 8]. The use of Doppler echocardiography to assess the change in cardiac outflow after this surrogate volume load provides an intuitive means of forecasting response to an administered fluid bolus. 

Modern intensive care is increasingly concerned with the avoidance of unnecessary invasive procedures which contribute to patient morbidity either directly or more often through the associated risk of catheter-related bloodstream infection [9]. 

Transoesophageal echocardiography may provide superior image quality in some cases and is increasingly utilised for cardiovascular monitoring on intensive care units. Nonetheless, it requires equipment, time, and skills that are less abundant on many intensive care units. It is contraindicated in some patients with upper airway or oesophageal surgery and also usually necessitates sedation which is not always achieved without adverse consequence. 

Accordingly, the objective of this review is to systematically evaluate the literature examining the use of transthoracic echocardiography in the assessment of dynamic markers of preload used to predict fluid responsiveness in the critically ill patient.

## 2. Methods

**Figure 2:**
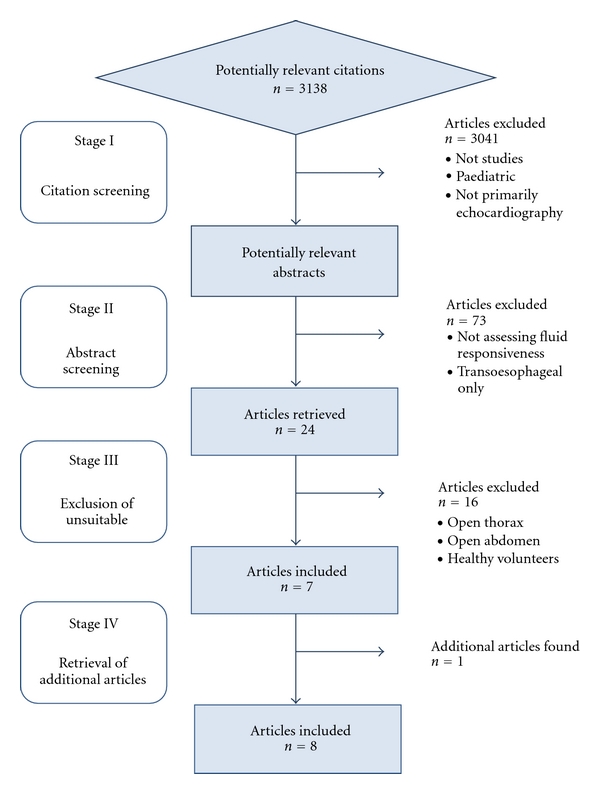
Citation filtering process.

An electronic literature search was carried out using Medline, EMBASE, CINAHL, and the Cochrane database of systematic reviews. The search terms used were ((fluid) OR (volume) OR (preload) OR (filling)) AND ((respons*) OR (status) OR (assess*)) AND ((echocardiograph*) OR (echog*)). The search was limited to “human” and “English language.” Figure 2 shows the process of filtering the studies selected for review.

Transoesophageal echocardiography studies were excluded, as were those in which the sample group, the equipment used and the reference test cut-off criteria implied the conclusions were not applicable to the critically ill patient in a high dependency of critical care environment.

### 2.1. Definitions of Fluid Responsiveness

“Fluid responsiveness” refers to a predefined rise in stroke volume or cardiac output after rapid fluid loading with a predetermined volume of fluid. Between investigators, there are inevitable differences in the choice of stroke volume or cardiac output, the volume of fluid given, the duration over which the fluid load was given, and the type of fluid given.

The diagnostic test or equivalent of the index test in these studies is defined as the echocardiographic test done to give a prediction of fluid responsiveness. In this review, this test will be termed the “predictive test.” The test done to assess the response to a fluid bolus once given is similar to a reference test but for the purposes of this review will be called the “response test.”

Responders are those patients in whom the cardiac output or stroke volume rises by the threshold amount after a given bolus of fluid.

### 2.2. Statistical Analysis

The Standards for Reporting of Diagnostic Accuracy (STARD) initiative developed a guide for assessing the quality of reporting of studies of diagnostic accuracy [10]. In this review, the STARD score was adapted to judge the quality of the investigation in each article selected (Table 1). A 19-point score was devised using 19 of the 25 STARD criteria. Each criterion was assigned one point and the overall score divided into categories: poor (score 0–10), adequate (11–15), and good (16–19).

The results of the selected studies were not meta-analysed due to the heterogeneity of methodologies, as well as the differences in patient selection, modes of ventilation and definition of fluid response. There was insufficient data for the construction of summary receiver-operator characteristic (SROC) curves or for the calculation of *Q* star statistics, and the simple average of sensitivity and specificity data is not an informative approach. Furthermore, the usage of a fixed-effects model such as SROC would be expected to produce exaggeratedly high levels of reported accuracy for a test that is to be put to use in the complex environment of the critically ill patient [11]. 

## 3. Results

Of the 3138 articles identified through the search terms, eight studies were included for review. The quality scores ranged between 13 and 15, indicating an adequate standard throughout. The studies were all small but of appropriate intensive care unit setting (Tables 2 and 3). 

### 3.1. Assessment of Fluid Responsiveness Using Transaortic Stroke Volume Increment to Passive Leg Raising

Five studies used transaortic stroke volume variation to predict fluid responsiveness using passive leg raising to mimic a fluid bolus [12–16]. Overall quality of the studies was adequate. All were done in intensive care patients with shock of various aetiologies. Three were carried out in medical intensive care units, one in a surgical unit, and the other in a mixed unit. Studies by Biais et al. [15], Lamia et al. [14], Maizel et al. [13], and Prèau et al. [12] included only patients with spontaneous respiratory effort, whether or not they were mechanically ventilated. 

**Figure 3:**
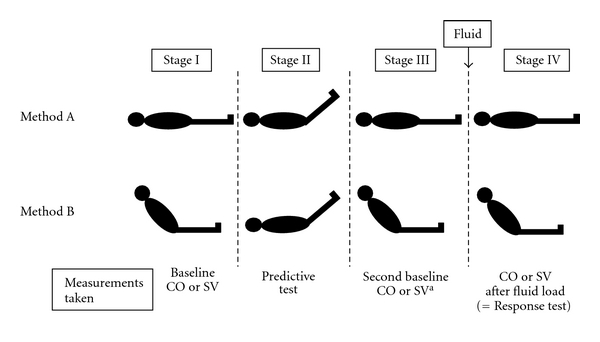
The stages of the two different methods of passive leg raising. CO cardiac output, SV stroke volume. ^a^Measurements at this stage were not taken in one study (Maizel).

Important differences between studies were evident in the study protocols. Maizel et al. [13] and Prèau et al. [12] used a 30 to 45 degree leg raise from the supine position where all others started with the patient semirecumbent at 30 to 45 degrees before tilting the bed until the patient was supine with legs raised (Figure 3). These two methods have been shown to result in different volumes of caudal surge of blood which potentially affects the validity of the test. Maizel et al. [13] had no second baseline measurement prior to fluid delivery. In all studies, the pretest baseline measurements of stroke volume were similar before the passive leg raise and before the assessment of a response to fluid bolus.

 All studies showed good sensitivity (77 to 100 percent) and specificity (88 to 99 percent) using a threshold of 10 to 15 percent increment of stroke volume or cardiac output.

Strikingly, stroke volume change with PLR predicted the correct response to volume expansion in 16 of the 18 patients with arrhythmia [16].

### 3.2. Assessment of Fluid Responsiveness Using Transaortic Stroke Volume Variation with Respiration

A single study by Biais et al. looked at the use of stroke volume variation for prediction of fluid responsiveness [19]. In this study, stroke volume variation measured across the aortic valve was used to predict a fluid response which was delivered as a 20 mL/kg/m^2^ bolus of 4% albumin. Stroke volume variation was calculated using the formula:

(1) $$\frac{{\text{SV}}_{\max}-{\text{SV}}_{\min}}{{\text{SV}}_{\text{mean}}}.$$

All patients were receiving mandatory ventilation and had no spontaneous respiratory effort.

The area under the receiver operator characteristic (ROC) curve was used to ascertain a threshold of nine percent stroke volume variation as being the most useful for discerning responders from nonresponders. Using this cut-off, there was excellent sensitivity and specificity (100 and 88 percent, resp.).

### 3.3. Assessment of Fluid Responsiveness through Respiratory Variation of IVC Diameter

Two studies by Barbier et al. and Feissel et al. used respiratory variation of the diameter of the IVC to predict fluid responsiveness [17, 18]. Both studies included only mechanically ventilated patients, without spontaneous respiratory effort. Each study compared the maximum and minimum diameter of the IVC just distal to the hepatic vein: *D*_max⁡_ and *D*_min⁡_, respectively (see Figure 1). Both studies expressed the distensibility of the IVC as a percentage index.

Barbier et al. used a “distensibility index” calculated by

(2) $$\frac{\left(D_{\max}-D_{\min}\right)}{D_{\min}},$$

whereas Feissel et al. corrected the mean of the two values:

(3) $$\frac{\left(D_{\max}-D_{\min}\right)}{0.5\left(D_{\max}+D_{\min}\right)}.$$

Barbier et al. showed a sensitivity and specificity of 90 percent using a cut-off distensibility index of 18 percent to indicate fluid responsiveness. Feissel et al. demonstrated a correspondingly high positive and negative predictive value, 93 and 92 percent, respectively, using an IVC diameter variation of 12 percent [18].

## 4. Discussion

This review shows that TTE is a highly discriminative test for the prediction of the stroke volume or cardiac output response to volume loading in critically ill patients, thus highlighting the potential for expansion of its role in quantitative assessment.

Importantly, TTE techniques appear useful in patients with spontaneous respiratory effort and those with arrhythmias: this is in contrast to many of the techniques that involve invasive monitoring which have been shown to be inaccurate in these situations [5].

Although TTE does not provide continuous monitoring which can be managed by nursing staff at the bedside, in reality, most clinical questions regarding fluid management arise intermittently. With equipment close at hand the time taken for a focussed TTE assessment rarely takes more than few minutes [20]. In addition, much of the data derived from pulmonary artery catheter measurement can be obtained using TTE, obviating the need for an invasive monitor that has been shown not to alter outcome [4].

The techniques of IVC diameter assessment, transaortic stroke volume variability with respiration and stroke volume increment with passive leg raising all provided strong predictive ability for response to a fluid bolus. The area under ROC curves was greater than 0.9 in all articles that presented the statistic. Although a clear threshold value for discriminating responders from nonresponders seems intuitively advantageous, clinicians are adept at coping with non-discriminatory results and using them to inform decisions made on the basis of the whole clinical picture.

None of the three TTE techniques is convincingly the best and if possible all three should be used to minimize the impact of their limitations. On occasion, this may not be achievable for a number of reasons. Local pain or delirium may preclude all or part of a TTE exam in a small minority of cases. In the 260 scans attempted within the studies selected, just 13 could not be performed for these reasons making this a well-tolerated procedure in the main. Thoracic or abdominal wounds may sometimes make views impossible to achieve. Obesity or rib prominence can also make TTE acoustic windows difficult to obtain but it is rare that at least a single usable view cannot be obtained in an individual. In the reviewed studies, only nine of the 260 attempted scans were abandoned due to difficulty with anatomy. Additionally, the applicable techniques will depend on the presence or absence of mechanical ventilation or dysrhythmias. For example, in a patient with atrial fibrillation who is fully ventilated, transaortic Doppler assessment is inaccurate but subcostal measurement of the IVC diameter variation can be safely used.

### 4.1. Clinical Application

The concept of “wet” and “dry” intensive care units has long been debated. The apparent benefits of goal-directed aggressive fluid resuscitation in the early stages of sepsis must be balanced with evidence for reduced morbidity when “restrictive” fluid regimes are used [21]. The literature lacks agreement on definitions of “wet” and “dry,” or “liberal” versus “restrictive” fluid protocols, and consequently, it is difficult to be certain of applicability to a particular setting. Brandstrup provided compelling evidence in colorectal surgical patients and the ARDSNET group in the subset of acute lung injury, but there is a paucity of further evidence [1, 22]. 

It is important to recognize that this review neither allows assumptions about the longevity of the response to fluid, nor the value of a continuous fluid infusion thereafter. It also follows that a forecast suggesting the patient will be fluid responsive in no way guarantees the safety of a delivered bolus in terms of increasing extravascular lung water or worsening regional organ oedema and function.

The literature contains a growing body of work on optimising haemodynamics using other echocardiographic parameters, beyond simple measures of contractility and structural pathology. Patterns of flow across the mitral valve and tissue velocity of the annulus have proved useful, principally when assessed in combination. Tissue velocity, particularly that measured close to the mitral valve annulus, assessed using Doppler imaging (TDI) provides an accurate estimation of diastolic function of the left ventricle irrespective of preload changes [23, 24]. Pulmonary artery occlusion pressure can be estimated by a number of methods, chiefly by tissue Doppler imaging but also by examining the pattern of movement of the interatrial septum [25]. Subtleties of the sonographic representation of interlobular septa can be used to assess extravascular pulmonary water and also correlate with pulmonary artery occlusion pressure [26]. An assessment using as many parameters as possible will provide valuable information at many stages of the patient's stay whether in managing the acute and unstable periods, or when weaning from the ventilator is troublesome [27].

Although detailed examination of the heart requires an experienced echocardiography practitioner, there is an increasing acceptance of the value of focussed echocardiographic assessments to answer common clinical questions arising in critical illness. This has arisen in tandem with the emergence of a number of courses and training programmes centred on evaluation of the critically ill patient by those less experienced in echocardiography. Jensen showed that with only limited training, a diagnostic transthoracic window was achieved 97 percent of the time when used in the evaluation of shock [20]. In the UK, a consultation process to provide a training template and curriculum for focussed echocardiography in critical care is currently underway [28].

### 4.2. Limitations

This review was restricted to the specific question of fluid response. In reality, echocardiographic assessment of the critically ill aims to gain as complete a picture as possible of the cardiovascular state. Ideally, this should also involve a full structural study in addition to inspection of left ventricular filling state and perhaps even ultrasonic examination of the lungs.

Furthermore, studies using transoesophageal echocardiography (TOE) were not selected for this review and, although it would seem intuitive that flow or diameter measurements techniques taken with one kind of echocardiography could be safely extrapolated to another, this ignores the differing technical restrictions of each technique. Transoesophageal echocardiography has its own growing evidence base for its application in intensive care and clearly where it is available provides invaluable haemodynamic information to inform clinical decisions.

A significant limitation of this review is the small size of the study groups since only a single study included more than 40 patients [16]; this is typical of studies of diagnostic accuracy. Meta-analysis was not performed, due to the heterogeneity of the methods and patient characteristics. In addition due to the similarity of the sensitivity and specificity data, it was felt that further statistical analysis would not add useful information.

It is conspicuous that only one article reported on the time between the initial predictive test and the subsequent assessment of a response to a fluid bolus [12]. Patients with haemodynamic instability can undergo rapid changes in cardiovascular parameters mandating that the period between the predictive and confirmatory tests should be as short as possible.

The amount of fluid used, the type used, and the rate at which it was given all impact upon the response test in these studies. Unfortunately, there is no agreed formulation for a standard fluid load although almost all studies use approximately the same formulation.

Although no specific details were given about the qualifications of the echocardiography operator or reader most studies inferred they were experienced. Furthermore, blinding of the operator or reader, to the measurements taken after volume loading was rare and this is, therefore, a source of observer bias within the data.

Intraobserver variability was considered by the majority of studies and attempts were made to measure it with variable success. An intuitively more useful measurement of reproducibility was achieved by examining the variability of repeated measurements of distensibility by Feissel et al. [18]. This showed a greater degree of intraobserver concordance at 3.4 percent. Any concern about the reproducibility of observations should however be viewed in the context of the consistent results achieved throughout the reviewed studies which is unlikely to have arisen by chance.

Of note, whilst the effects of varying tidal volumes on echocardiographic parameter assessment are minimal, the impact of raised intra-abdominal pressure and of different positive end expiratory pressure is largely unstudied [29].

### 4.3. Future Developments

The clinical question that was not addressed in any of the articles was that of the “real-world” value of echocardiographic approaches to assessing fluid responsiveness. The studies reviewed do not provide us with information about translation into effects on morbidity or mortality, nor is there yet such a current evidence base in the literature. This evidence may well originate in the context of future investigation into the dilemma of conservative versus liberal fluid management.

Transpulmonary microsphere contrast has already been shown to dramatically improve volumetric assessment and its use in the critically ill would intuitively improve the clinical utility of the modality still further [30]. Three-dimensional echo remains in its infancy within the intensive care unit but the promise of increased, automated volumetric accuracy, and improved diagnostic clarity will also undoubtedly be examined in the near future [31, 32].

## 5. Conclusion

Transthoracic echocardiography is becoming a powerful noninvasive tool in the daily care of the critically ill. This review brings together the evidence for employing TTE to predict fluid responsiveness. Assuming there is equipment and local expertise TTE is a repeatable and reliable method of predicting volume responsiveness in the critically ill.

Transaortic stroke volume variation with the respiratory cycle, stroke volume difference following passive leg raising, and IVC diameter changes with respiration all provide good prediction of the likelihood of a response to a fluid bolus. The techniques can be used individually to address the needs of different patients and in combination to triangulate clinical information where uncertainties may occur.

The studies reviewed form a robust platform of physiological data on which to base further studies involving larger numbers of patients which engage with clinically relevant outcomes, such as inotrope use, blood pressure, length of stay, and time to weaning from mechanical ventilation.

Improved access to clinician-echocardiographers through a defined training process will facilitate such clinical studies and give patients access to accurate noninvasive information in answer to the daily clinical conundrum of fluid responsiveness.

## Figures and Tables

**Table 1:** Modified STARD criteria assessment [10].

Criteria	Specific question
1	Was the study population described (inclusion and exclusion criteria included)?
2	Is there a description of the sampling (e.g., consecutive patients, if not why not?)?
3	Is it clear whether the tests were done prospectively or retrospectively?
4	Is there a description of the response test (including fluid bolus)?
5	Is there a detailed description of the equipment and techniques used in the tests?
6	Is the rationale for cut-offs and ranges given?
7	Is there detail of the operators in terms of number and training?
8	Is there detail of what information was available to the readers of the response ?
9	Were the statistical methods for comparing diagnostic accuracy detailed?
10	Are there details of tests of reproducibility?
11	Are the patient demographics and comorbidities shown?
12	Is there detail of those meeting inclusion criteria but not undergoing either test?
13	Was there detail of the interval between predictive and response tests?
14	Is there a report cross-tabulating predictive and response test results?
15	Is diagnostic accuracy described, including likelihood ratios or data to calculate them?
16	Is there mention of how missing values were dealt with (i.e., unobtainable values)?
17	Are the estimates of accuracy variability between operators/readers included?
18	Are there estimates of reproducibility?
19	Is the clinical applicability of the study findings discussed?

**Table 2:** Characteristics of studies selected.

Study	Technique	Patient group	Selection	Ventilation	Rhythm	Volume and type	Time (min)	Response criteria
Barbier et al. [17]	IVC DI	Mixed ICU	Shock (sepsis) and acute lung injury	All mand	Any	7 mL/kg colloid	30	>15% CO TTE
Feissel et al. [18]	Δ*D*_IVC_	Medical ICU	Shock (sepsis)	All mand	Any	8 mL/kg colloid	20	>15% CO TTE
Lamia et al. [14]	PLR	Medical ICU	Shock (sepsis or hypovolaemia)	All spont	Regular SR, or AF	500 mL crystalloid	15	>15% SV TTE
Maizel et al. [13]	PLR	Mixed ICU	Shock (unspecified)	All spont	Regular SR	500 mL crystalloid	15	>12% CO TTE
Biais et al. [15]	PLR	Surgical ICU	Shock (sepsis or haemorrhage)	All spont	Any	500 crystalloid	15	>15% SV TTE
Biais wt al. [19]	SVV	Surgical ICU	Post-operative (liver surgery)	All mand	Regular SR	20 mL/kg/m^2^ colloid	20	>15% CO TTE
Thiel et al. [16]	PLR	Medical ICU	Shock (unspecified)	Mixed	Any	500 mL crystalloid or colloid	Unspec	>15% SV TTE
Préau et al. [12]	PLR	Medical ICU	Shock (sepsis or acute pancreatitis)	All spont	Regular SR	500 mL colloid	<30	>15% SV TTE

Selection: inclusion criteria summary, PLR: passive leg raising, spont: spontaneous respiratory effort whether or not on mechanical ventilation, mand: ventilator giving mandatory breaths only and patient fully adapted to ventilator, SR: sinus rhythm, AF: atrial fibrillation, TTE: transthoracic echocardiography, SV: stroke volume, CO: cardiac output, Δ*D*_IVC_ change in IVC diameter adjusted by the mean (see text), IVC DI: IVC distensibility index (see text), and unspec: unspecified time.

**Table 3:** Collated results of all included studies.

Study	Number of tests	Predictive test	Threshold	Resp %	Intra-obs %	Inter-obs %	AUC (ROC)	Sens	Spec	PLiR	NLiR	PPV	NPV	*r*
Lamia et al. [14]	24	PLR SVI or CO rise	≥12.5%	54	2.8 ± 2.2	3.2 ± 2.5	0.96 ± 0.04	77	99	77	0.23			0.79
Maizel et al. [13]	34	PLR CO rise	≥12%	50	4.2 ± 3.9	6.5 ± 5.5	0.90 ± 0.06	63	89	5.73	0.42	85	76	0.75
		PLR SV rise	≥12%		4.2 ± 3.9	6.2 ± 4.2	0.95 ± 0.04	69	89	6.27	0.35	83	73	0.57
Biais et al. [15]	34	PLR SV rise	≥13%	67		SI	0.96 ± 0.03	100	80	5.00	0.00			
Thiel et al. [16]	102	PLR SV rise	≥15%	46		SI	0.89 ± 0.04	81	93	11.57	0.20	91	85	
Préau et al. [12]	34	PLR SV rise	≥10%	41		SI	0.90 ± 0.04	86	90	8.60	0.16	86	90	0.74
		PLR dVF rise	≥8%				0.93 ± 0.04	86	80	4.30	0.18	75	89	0.58

Biais et al. [15]	30	SVV	≥9%	47		SI	0.95	100	88	8.33	0.00			0.80

Barbier et al. [17]	23	IVC DI	≥18%	41	8.7 ± 9	6.3 ± 8	0.91 ± 0.07	90	90	9.00	0.11			0.90
Feissel et al. [18]	39	Δ*D*_IVC_	≥12%	41	3 ± 4	SI						93	92	0.82

Threshold: cut-off between responders and nonresponders, Resp: proportion responding to fluid load, Intra-obs: intraobserver variability, Inter-obs: interobserver variability, AUC(ROC): area under the receiver-operator curve, Sens: Sensitivity, Spec: Specificity, PLiR: positive likelihood ratio, NLiR: negative likelihood ratio, PPV: positive predictive value, NPV: negative predictive value, *r*: correlation coefficient, PLR: Passive leg raising, SI: single investigator/reader, CO: cardiac output, SV: stroke volume, dVF: change in femoral artery velocity as measured by Doppler, SVI: stroke volume index, LVEDAI: left ventricular end-diastolic area, *E*/*E*_*a*_: mitral *E*-wave velocity/mitral annulus *E* velocity measured by tissue Doppler, Δ*D*_IVC_: change in IVC diameter (*D*) as calculated by (*D*_max⁡_ − *D*_min⁡_)/0.5(*D*_max⁡_ + *D*_min⁡_), IVC DI: IVC distensibility index calculated by (*D*_max⁡_ − *D*_min⁡_)/*D*_min⁡_.
